# Editorial: Biomarker-guided strategies in NSCLC immunotherapy

**DOI:** 10.3389/fonc.2025.1769857

**Published:** 2026-01-09

**Authors:** Myrto K. Moutafi, Kok Haw Jonathan Lim, Panagiota Economopoulou, Giannis Mountzios

**Affiliations:** 1 University General Hospital Attikon, Athens, Greece; 2 Faculty of Biology Medicine and Health, The University of Manchester, Manchester, United Kingdom; 3 Advanced Immunotherapy and Cell Therapy Team, The Christie National Health Service (NHS) Foundation Trust, Manchester, United Kingdom; 4 4th Oncology Clinic and Clinical Trials Department, Henry Dunant Hospital Center, Athens, Greece

**Keywords:** biomarker, immune checkpoint inhibitor, NSCLC, PD-L1, tumor infiltrate lymphocyte

Immunotherapy has reshaped the clinical landscape of non-small cell lung cancer (NSCLC), establishing immune checkpoint blockade (ICB) as a backbone of treatment for advanced disease (1). Nonetheless, only a subset of patients achieves durable benefit, while many exhibit primary resistance or relapse after initial response (2, 3). These challenges underscore an urgent need for reliable biomarkers that can guide treatment selection, predict outcomes, and illuminate mechanisms of resistance. In this Research Topic, Biomarker-Guided Strategies in NSCLC Immunotherapy, seven diverse contributions, including original research, machine-learning innovations, meta-analysis, and genetic epidemiology, collectively advance biomarker discovery and application in NSCLC. Together, these studies provide a timely overview of the current biomarker landscape, highlighting both the opportunities and limitations of deploying biomarker-guided strategies to individualize immunotherapy.

A central theme across multiple contributions is the complexity and heterogeneity of PD-L1 expression, long the most widely used biomarker for ICB selection. Zhou et al. investigated subregional PD-L1 expression within primary tumors in advanced NSCLC patients treated with ICB-based regimens. Their study demonstrates that PD-L1 is not uniformly expressed throughout the tumor mass and that spatial heterogeneity correlates with clinical outcomes. These findings highlight that reliance on a single biopsy or a single PD-L1 threshold may overlook biologically meaningful variation. As PD-L1 remains the most frequently used clinical biomarker, the work by Zhou et al. urges deeper consideration of sampling strategies and suggests that quantitative and spatial PD-L1 features could refine existing predictive models.

At the same time, biomarker research continues to expand far beyond PD-L1. Rother et al. offer a comprehensive overview of biomarkers associated with immunotherapy resistance in NSCLC, synthesizing tumor-intrinsic mechanisms such as genomic instability, oncogenic drivers, and antigen presentation deficits with tumor-extrinsic influences including immune cell infiltration, cytokine signaling, and systemic inflammation. Their review underscores the growing consensus that single biomarkers are insufficient and that multi-parametric signatures incorporating both tumor biology and immune contexture will be required to more accurately predict resistance and guide future combination strategies.

Genomic biomarkers remain critical in NSCLC, particularly as oncogene-driven tumors often exhibit reduced responsiveness to immunotherapy. To address this challenge, Hao et al. employed an integrative machine-learning framework combining 3D pretrained ConvNeXt modeling, radiomics features, and clinical parameters to non-invasively predict EGFR mutation subtypes. Their approach highlights the growing utility of radiomics and deep learning as scalable tools for molecular characterization when tumor tissue is limited or when repeated sampling is impractical.

The interplay between environmental exposures and tumor biology is another emerging area of biomarker relevance. Cao et al. used Mendelian randomization and mediation analyses to dissect the causal relationships between diverse smoking behaviors, potential therapeutic targets, and NSCLC risk. They identify three potential molecular mediators (NCAPD2, IL11RA, and MLC1). Notably IL11RA mediates about 22.2% of the effect of past smoking on NSCLC risk. Integrating bioinformatics with MR techniques can pinpoint novel therapeutic targets.

Systemic inflammation is increasingly recognized as a determinant of immunotherapy outcomes. In this context, Wu et al. conducted a meta-analysis of the modified Glasgow Prognostic Score (mGPS) in NSCLC patients receiving ICB. They demonstrated that elevated mGPS, reflecting elevated C-reactive protein and hypoalbuminemia, is associated with inferior outcomes across multiple studies. As a simple, widely available clinical metric, mGPS may offer a practical tool to stratify patients and guide prognostication in real-world ICB settings, where access to advanced molecular testing may vary.

A distinct contribution comes from the work of Dan et al., who investigated PD-1 expression within tumor-infiltrating lymphocytes (TILs) in early-stage NSCLC. While PD-1 expression on tumor cells or circulating cells has been studied extensively, their focus on TIL-specific PD-1 adds new dimension by linking immune checkpoint expression directly to the local immune microenvironment. They report that PD-1 expression patterns within TILs are prognostically significant, suggesting that immune contexture, and not only tumor-centric biomarkers, is critical for understanding clinical outcomes, even in early-stage disease.

Completing the Research Topic, Zhang et al. examined PD-L1 expression and clinical outcomes in ROS1-rearranged NSCLC, a molecularly defined subgroup traditionally treated with targeted therapies such as crizotinib. Their findings show that PD-L1 expression is variable in ROS1-rearranged tumors and may correlate with treatment outcomes, raising important questions about how immunotherapy might be integrated with targeted therapy in select patients. As rare oncogenic drivers become increasingly characterized, subgroup-specific biomarker data such as that presented by Zhang et al. will be essential to informing personalized approaches.

These studies collectively highlight several overarching themes. First, biomarker biology in NSCLC is multifactorial and cannot be distilled into a single variable. Spatial heterogeneity of PD-L1 expression, as shown by Zhou et al., and immune-microenvironmental features, such as PD-1-positive TILs demonstrated by Dan et al., speak to the importance of contextualizing biomarkers within the tumor ecosystem. Second, multi-modal biomarker strategies, integrating genomics, imaging, machine learning, systemic inflammation, and environmental exposures, are increasingly recognized as necessary to capture the complexity of ICB response. Third, the boundaries of biomarker innovation continue to expand through computational approaches, genetic epidemiology, and immune profiling.

As immunotherapy becomes more deeply embedded in the therapeutic landscape of NSCLC, future work will need to focus on harmonizing biomarker platforms, validating combinatorial models in prospective studies, and integrating biomarker-guided approaches into clinical trial design (4, 5). In parallel, translation of machine-learning and radiomics-based biomarkers into routine practice will require standardization, transparent validation, and cross-platform reproducibility.

In summary, the contributions in this Research Topic collectively illuminate the complexity and promise of biomarker-guided strategies in NSCLC immunotherapy. They reinforce that precision immunotherapy will require integrative approaches that reflect tumor genomics, immune ecology, host physiology, and environmental exposures. Together, these seven studies chart an important path toward more personalized, predictive, and effective immunotherapy for patients with NSCLC.

## References

[1] Leena G Delvys R-A Shirish G Emilio E Enriqueta F DA FláChecktae . Pembrolizumab plus chemotherapy in metastatic non–small-cell lung cancer. New Engl J Med. (2018) 378:2078–92. doi: 10.1056/NEJMoa1801005, PMID: 29658856

[2] Kang K Liu S Yao Z Xue J Lu Y . Addressing clinical needs in NSCLC immunotherapy: Mechanisms of resistance and promising combination strategies. Cell Rep Med. (2025) 6. doi: 10.1016/j.xcrm.2025.102315, PMID: 40876451 PMC12490215

[3] Schoenfeld AJ Hellmann MD . Acquired resistance to immune checkpoint inhibitors. Cancer Cell. (2020) 37:443–55. doi: 10.1016/j.ccell.2020.03.017, PMID: 32289269 PMC7182070

[4] Lu T Hamidi H Socinski MA Reck M Cappuzzo F Barlesi F . Non-small cell lung cancer molecular subtypes and vulnerability to immunotherapy treatment combinations. Nat Commun. (2025). doi: 10.1038/s41467-025-66803-8, PMID: 41339316 PMC12775477

[5] Lim KHJ Tippu Z Corrie PG Hubank M Larkin J Lawley TD . MANIFEST: Multiomic Platform for Cancer Immunotherapy. Cancer Discovery. (2025) 15:878–883. doi: 10.1158/2159-8290.CD-25-009, PMID: 40313120

